# 512 Murine Tissue-Resident F4/80+ Macrophages Exhibit Transcriptomic Immune Reprogramming Chronically Following Burn Injury

**DOI:** 10.1093/jbcr/iraf019.141

**Published:** 2025-04-01

**Authors:** Denise Hernandez, Han Kim, Madelyn Bucci, Micah Willis, Maisie Mortlock, Shannon Wallet, Robert Maile

**Affiliations:** University of Florida; University of Florida; University of Florida; University of Florida; University of Florida; University of Florida; University of Florida

## Abstract

**Introduction:**

Burn injuries are among the most serious forms of trauma and are associated with high morbidity and mortality. Patient clinical outcomes are inextricably linked with immune and metabolic function with clear clinical phases. Early onset Systemic Inflammatory Response Syndrome (SIRS), often progresses into a chronic phase of Persistent Inflammation immunosuppression, and Catabolism Syndrome (PICS). Contrary to the clinical picture, we and others have demonstrated that issue resident innate cells isolated late 14 days after burn injury are hyper-responsive to further immune stimulation ex vivo. We hypothesize that chronic clinical outcomes observed after burn injury are a consequence of long-term altered innate immune reprogramming.

**Methods:**

Wildtype female C57BI/6 mice weighing 18-22g underwent a 20% total body surface area full-thickness cutaneous contact burn or sham injury (n=6 per group). Spleens were collected at acute (2d), sub-chronic (9d), and chronic (14d) time points; we positively selected for F4/80+ tissue resident macrophages (trMø) using magnetic bead cell separation (MACS, Miltenyi Biotec Inc). RNA was subsequently purified from lysed F4/80+ trMø and used to perform nanoString immune and metabolic gene transcriptomic analysis (Immune Profiling Panel and Metabolic Pathways Panel CodeSets) with corresponding Ingenuity Pathway Analysis (IPA).

**Results:**

Analysis revealed significant gene expression changes in each burn group compared to sham mice, and between mice 14d versus 2d following injury. For example, F4/80 macs at day 2, 7 and 14 after burn injury still exhibit significant (p< 0.05) up and down-regulation of a broad range of immune and metabolic genes compared to uninjured sham mice. IPA showed multiple signaling pathways were significantly (p < 0.05) impacted, such as an increase in IL-10 signaling, glycolysis (mainly via increased expression of hexokinases and glucose-6-phosphatase genes), and OXPHOS pathway Z-scores in burn mice at all time points compared to sham. Notable changes include HIF1α and LAT. In contrast to 2d, 14d post-burn TRMø exhibited a significant upregulation of various pro-inflammatory gene such as IFNA1, various chemokines and cytokines including CCL2, IL25, IL12, and TRAF4.

**Conclusions:**

These data indicate that long term immune gene reprogramming may be a key driving mechanism of immune dysfunction after severe burn injury, in our murine models. We are correlating these findings to our previous epigenetic findings in these cells to confirm that TRMø are affected by post-burn genetic modification.

**Applicability of Research to Practice:**

Given the chronic nature of burn immune dysfunction, it is important to define mechanisms to identify therapeutics and identify biomarkers to predict PICS.

**Funding for the Study:**

NIH NIGMS R01 GM 146134

